# Proteome Analysis of Potato Starch Reveals the Presence of New Starch Metabolic Proteins as Well as Multiple Protease Inhibitors

**DOI:** 10.3389/fpls.2018.00746

**Published:** 2018-06-15

**Authors:** Stanislas Helle, Fabrice Bray, Jérémy Verbeke, Stéphanie Devassine, Adeline Courseaux, Maud Facon, Caroline Tokarski, Christian Rolando, Nicolas Szydlowski

**Affiliations:** ^1^Univ. Lille, CNRS, UMR8576 – UGSF – Unité de Glycobiologie Structurale et Fonctionnelle, Lille, France; ^2^Univ. Lille, CNRS, USR 3290 – MSAP – Miniaturisation pour la Synthèse, l’Analyse et la Protéomique, Lille, France

**Keywords:** starch, potato, proteomics, starch-binding protein, starch synthase, *Solanum tuberosum*

## Abstract

Starch bound proteins mainly include enzymes from the starch biosynthesis pathway. Recently, new functions in starch molecular assembly or active protein targeting were also proposed for starch associated proteins. The potato genome sequence reveals 77 loci encoding starch metabolizing enzymes with the identification of previously unknown putative isoforms. Here we show by bottom-up proteomics that most of the starch biosynthetic enzymes in potato remain associated with starch even after washing with SDS or protease treatment of the granule surface. Moreover, our study confirmed the presence of PTST1 (Protein Targeting to Starch), ESV1 (Early StarVation1) and LESV (Like ESV), that have recently been identified in Arabidopsis. In addition, we report on the presence of a new isoform of starch synthase, SS6, containing both K-X-G-G-L catalytic motifs. Furthermore, multiple protease inhibitors were also identified that are cleared away from starch by SDS and thermolysin treatments. Our results indicate that SS6 may play a yet uncharacterized function in starch biosynthesis and open new perspectives both in understanding storage starch metabolism as well as breeding improved potato lines.

## Introduction

Starch is a storage form of carbohydrates in plants and the main source of calories in human and animal diets. Moreover, this polymer is used in many industrial applications for food and non-food purpose. Amylose and amylopectin, the two polymers composing starch, are made of α-1,4- and α-1,6-bound glucose residues (Buléon et al., 1998). The former is a rather linear molecule containing about 1% of α-1,6 linkages while the latter is moderately branched with 5–6% of α-1,6 linkages. Potato starch contains on average 0.06% of proteins on a weight basis (Jobling, 2004). To date, most of the identified starch-bound proteins are starch metabolic enzymes (Grimaud et al., 2008).

Granule Bound Starch Synthase 1 (GBSS1) elongates the α-1,4 glucans composing amylose (Ball et al., 1998). GBSS1 represents up to 95% of the granule bound proteins and is only fully active under this granule-associated form (Rongine De Fekete et al., 1960). On the other hand, amylopectin is synthesized by the concerted activities of soluble starch synthases (SSs), starch branching enzymes (BEs) and starch debranching enzymes (DBEs). To our knowledge, among the SS isoforms, only SS1, SS2 and SS3 were identified by proteomic analysis of maize starch as well as in starches from potato, rice, pea, barley and wheat (Denyer et al., 1993; Peng et al., 2000; Borén et al., 2004; Grimaud et al., 2008; Stensballe et al., 2008; Xing et al., 2016). A series of evidence show that SS4 is involved in starch initiation although the precise mechanism is still under investigation (Roldan et al., 2007; Szydlowski et al., 2009; Raynaud et al., 2016; Seung et al., 2016). However, the enzyme was observed at the edge of starch granules by confocal microscopy analysis of Arabidopsis leaf chloroplasts and potato amyloplasts (Szydlowski et al., 2009; Gámez-Arjona et al., 2011). A fifth SS isoform, SS5, is phylogenetically related to SS4 but lacks the C-terminal X-X-G-G-L motif conserved within other SSs (Deschamps et al., 2008; Liu et al., 2015). The corresponding gene is conserved in plants (Deschamps et al., 2008) and is expressed during grain filling in maize (Liu et al., 2015). Thus, a function in starch synthesis was proposed but it remains to be investigated (Liu et al., 2015). An additional putative isoform, SS6 was found in the potato genome (Yu et al., 2012; Van Harsselaar et al., 2017). The corresponding gene displays a specific dynamic expression profile during tuberization (Yu et al., 2012). The putative function of this isoform in starch biosynthesis as well as its repartition within the plant kingdom is unknown.

In potato, two isoforms of branching enzymes, BE1 and BE2, which introduce α-1,6 linkages within amylopectin molecules, have been characterized (Schwall et al., 2000; Rydberg et al., 2001). Similar to SSs, starch branching enzymes were found in association with the starch granule in numerous plant species (Mu-Forster et al., 1996; Borén et al., 2004; Regina et al., 2005; Umemoto and Aoki, 2005; Grimaud et al., 2008). Interestingly, wheat BE2b interacts with SS1 and SS2a to form a trimeric complex associated with the granule (Tetlow et al., 2004, 2008b). Another complex including BE2b, BE1 and PHS1 (Starch Phosphorylase 1) was also identified although it was associated with starch only in a *be2b* mutant background (Tetlow et al., 2004; Liu et al., 2009; Subasinghe et al., 2014). Debranching enzymes comprise isoamylases and pullulanase. Both hydrolyze the α-1,6 bonds of amylopectin (Wattebled et al., 2005, 2008). Plants contain one pullulanase (PU) and three isoamylases (ISA1, ISA2 and ISA3). ISA1 and ISA2 participate in starch synthesis and interact to form hetero and homo complexes where the catalytic activity is carried by ISA1 (Delatte et al., 2005). DBEs seem to be predominantly soluble but ISA2 and PU were recently identified in association with starch in rice (Xing et al., 2016; Yu and Wang, 2016).

A set of enzymes (i.e., GWD, PWD, LSF1, LSF2 and SEX4) participate in starch breakdown via glucan phosphorylation/dephosphorylation (Ritte et al., 2000; Kötting et al., 2005; Comparot-Moss et al., 2010; Hejazi et al., 2010; Santelia et al., 2011). GWD (Glucan Water Dikinase) and PWD (Phosphoglucan Water Dikinase) phosphorylate starch at C6- and C3-position of the glucose residues, respectively (Ritte et al., 2006). GWD was observed in internal association with purified potato starch granules while PWD was shown to bind the surface of starch granules in Arabidopsis (Ritte et al., 2000; Kötting et al., 2005). LSF2 (Like SEX Four 2) and SEX4 (Starch Excess 4) also bind to starch granules as demonstrated both *in vitro* and with native starch granules isolated from Arabidopsis (Santelia et al., 2011). On the other hand, LSF1 (Like SEX Four 1) is likely associated with the granule surface according to the suborganellar distribution of the corresponding GFP-tagged proteins in Arabidopsis protoplasts (Comparot-Moss et al., 2010). Noteworthy, apart from GWD that appears to be entrapped in the starch matrix, these enzymes are located at the surface of the granules, consistent with the current model for phosphorylation/dephosphorylation driven starch breakdown (Silver et al., 2014).

In addition to starch metabolic enzymes, a series of proteins devoid of known catalytic domains were recently identified (Peng et al., 2014; Seung et al., 2015; Feike et al., 2016). Floury Endosperm 6 (FLO6) and Protein Targeting to Starch (PTST1) both contain a CBM48 (Carbohydrate Binding Module 48) that drives protein binding to starch (Peng et al., 2014; Seung et al., 2015). PTST1 also binds to GBSS1 and was proposed to target the amylose-synthesizing enzyme to starch polysaccharides (Seung et al., 2015). On the other hand, FLO6 interacts with ISA1 and is likely regulating its binding to starch although the exact mechanism remains to be uncovered (Peng et al., 2014). In both cases, inactivation of the corresponding gene leads to a phenotype similar to those of *gbss1* and *isa1* mutants, respectively (Peng et al., 2014; Seung et al., 2015). Furthermore, Early Starvation 1 (ESV1) and its homolog Like ESV1 (LESV) do not display any characterized domain (Feike et al., 2016). Both proteins are involved in the regulation of starch breakdown and likely play antagonistic roles (Feike et al., 2016). The molecular mechanisms underlying these functions are still under investigation. Nevertheless, it was proposed that both proteins modulate the organization of starch glucans and consequently affect their accessibility to catabolic enzymes (Feike et al., 2016).

These recent investigations highlight that non-catalytic starch binding proteins can also be involved in starch metabolism as well as its regulation and that some minor proteins remain to be characterized. In consequence, exhaustive proteomic analysis of starch is likely to lead to the identification of yet unknown functions in starch metabolism. The nuclear genome of a homozygous doubled-monoploid potato clone has been sequenced (Xu et al., 2011). *In silico* analysis revealed 77 genomic loci encoding enzymes related to starch metabolism with numerous novel putative isoforms (Van Harsselaar et al., 2017). Based on the potato genome annotation, the starch bound proteins were analyzed by mass spectrometry in this study. In addition to the already known starch metabolizing enzymes, we report on so far non-described starch bound proteins that are likely involved in the metabolism. Most strikingly, these include a novel isoform of starch synthase, SS6 that contains both K-X-G-G-L and X-X-G-G-L motifs, and a series of protease inhibitors. The latter are associated with the granule surface and are removed by SDS or thermolysin treatments.

## Materials and Methods

### Plant Material and Starch Isolation

Starch granules were isolated from tubers of *Solanum tuberosum* (Mona Lisa) cultivated in the field in Villeneuve d’Ascq (50.607735, 3.143431), France, between March and July 2014. Potato tubers were washed with tap water and peeled prior extraction. Tubers were ground with a blender in 200 mL of ultrapure water. Tuber extracts were then filtered through a nylon net (100 μm mesh) and left for sedimentation of starch granules for 3 h. The supernatant was then removed and sedimented starch was resuspended in 500 mL of ultrapure water. Starch suspensions were subsequently washed three times with 1 L of ultrapure water and stored in 20% ethanol at 4 °C.

### Surface Treatment of Starch and Protein Extraction

300 mg of starch granules were either washed five times with 2 mL of 2% SDS or submitted to thermolysin treatment. 60 μg of thermolysin (Promega, France) were added to 300 mg of starch in 50 mM Tris, 0.5 mM CaCl_2_, pH 8.0 prior to incubation overnight at 37°C. Starch granules were then washed five times with 2 mL of ultrapure water and protein extraction was carried out with the addition of 5 mL of extraction buffer (0.2 M Tris, 2% SDS, 20% glycerol, 50 mM DTT, pH 6.8) and subsequent incubation at 100°C for 20 min with regular vortexing. After centrifugation (20000 *g*, 10 min), the supernatant was concentrated on an ultrafiltration column (Amicon Ultra-15, Merck, Germany) by centrifugation for 30 min at 7500 *g*. Bottom-up identification was then performed either after protein separation by SDS-PAGE or by shotgun proteomics.

### Sodium Dodecyl Sulfate Polyacrylamide Gel Electrophoresis (SDS-PAGE)

All chemicals were purchased from Sigma-Aldrich (St. Louis, MO, United States). A stock solution of acrylamide (50 mL) was prepared using 14.6 g acrylamide and 390 mg *N*,*N*,*N*′,*N*′ methylene-bis-acrylamide as a cross-linking agent. 330 mg of Dextran (MW 500 000) was also incorporated to the previous solution in order to improve gel separation according to the protocol developed in (Ainseba-Chirani et al., 2011). The polyacrylamide concentrations were 4% and 7 or 10% in stacking (0.5 M Tris-Base, 14 mM SDS, pH 6.8) and running (1.1 M Tris-Base, 0.4 M Tris-HCl and 20 mM SDS, pH 8.8) gels, respectively. Protein samples were dissolved in a denaturing buffer containing 0.375 M Tris, 2 mM DTT, 1.4 M SDS, 2% glycerol (v/v), and 0.02% bromophenol blue (w/v). Low- and high-molecular weight (LMW and HMW, respectively) protein markers were used for gel calibration. Electrophoresis was carried out using a SE 600 Ruby system (GE Lifescience, Velizy-Villacoublay, France) at 40 mA per gel in a migration buffer composed of 0.025 M Tris, 0.192 M glycine, 0.1% SDS (w/v). Gels were fixed in a 20% ethanol, 7% acetic acid (v/v) solution for 20 min prior to overnight incubation in a Ru(BPS)_3_-containing staining solution under continuous gentle agitation. Gels were finally washed in 25% ethanol, 14% (v/v) acetic acid and digitized using a Typhoon^®^ 9000 scanner (GE Lifescience, Velizy-Villacoublay, France). Image acquisition was performed at a resolution of 100 nm, photo-multiplicators 580 V with the blue laser and filters corresponding to *l*_Exc_ = 488 nm for excitation ad *l*_Em_ = 610 nm for emission.

### In-Gel Tryptic Digestion

The protein bands were excised from the gel and destained in 50% acetonitrile, 25 mM NH_4_HCO_3_ prior to dehydration in 100% acetonitrile. Proteins were reduced by incubating the gel bands in 10 mM DTT, 25 mM NH_4_HCO_3_ at 56°C for 1 h, and alkylation was performed with 55 mM iodoacétamide, 25 mM NH_4_HCO_3_ at room temperature for 45 min in the dark. Gel pieces were dehydrated in 50% acetonitrile, 25 mM NH_4_HCO_3_ twice for 30 min, and then in 100% acetonitrile during 5 min. Gel samples were dried for 30 min at room temperature prior to rehydration with a solution of sequencing-grade trypsin (10 ng/μl in 25 mM NH_4_HCO_3_) on ice for 30 min and subsequently submitted to overnight incubation at 37°C. The resulting peptides were extracted with 100% acetonitrile containing 0.1% trifluoroacetic acid (TFA) for 30 min. The extracts were finally dried in a vacuum concentrator and dissolved in a solution of 0.1% formic acid for mass spectrometric analysis.

### Enhanced Filter Aided Sample Preparation (eFASP) Tryptic Digestion

Enhanced Filter Aided Sample Preparation protocol was performed following the protocol of (Erde et al., 2014), with some modifications. Protein extracts were incubated in 50 μL of reducing buffer (4% SDS, 0.2% deoxycholic acid, 50 mM DTT, 200 mM NH_4_HCO_3_) overnight at 4°C prior to centrifugation at 13,000 *g* for 15 min. The supernatants were then mixed with 200 μL of exchange buffer (8 M urea, 0.2% deoxycholic acid, 100 mM NH_4_HCO_3_, pH 8) and transferred on ultrafiltration units (Amicon^®^, 10 kDa cutoff; Millipore, Billerica, MA, United States). The latter were centrifuged at 13000 *g* during 30 min and an additional buffer exchange step was performed. Reduced proteins were alkylated within the filtration units with the addition of 100 μL of 8 M urea, 50 mM iodoacetamide, 100 mM NH_4_HCO_3_, pH 8 followed by incubation at 37°C at room temperature in darkness. Proteins were then washed once with 200 μL of exchange buffer and twice with 200 μL of eFASP digestion buffer (50 mM NH_4_HCO_3_, 0.2% deoxycholic acid pH 8). Tryptic digestion was carried out by incubating protein samples with 1 μg of trypsin in 120 μL of eFASP digestion buffer at 37°C for 16 h and under constant agitation. Peptides were recovered in a new collection tube by centrifugation at 13000 *g* for 20 min. For complete peptide recovery, ultrafiltration units were subsequently washed twice with 50 μL of 50 mM NH_4_HCO_3_.

### Phase Transfer

Peptides were precipitated with the addition of 200 μL of ethyl acetate and 2.5 μL of TFA. Peptide precipitates were then washed three times with 800 μL of ethyl acetate with centrifugation at 13000 *g* for 10 min after each wash. Ethyl acetate was evaporated by placing the peptide pellets at 60°C, in a fume hood, for 5 min and residual organic and volatile salts were removed by vacuum drying. This vacuum drying step was then repeated twice after the addition of 50% methanol and dried pepetides were dissolved in 10 μL of milliQ water. One microliter of peptide solution, corresponding to 5 μg of protein digest, was diluted in 9 μL of nano-HPLC buffer (5% acetonitrile and 0.1% formic acid).

### Protein Identification Using LC-MS/MS

A nanoflow HPLC instrument (U3000 RSLC Thermo Fisher Scientific^TM^) was coupled on-line to a Q Exactive plus (Thermo Scientific^TM^) with a nanoelectrospray ion source. 1 μL of gel-band peptide extracts or 1 μg of eFASP digests were loaded onto the preconcentration trap (Thermo Scientific^TM^, Acclaim PepMap100 C18, 5 μm, 300 μm i.d × 5 mm) using partial loop injection, for 5 min at a flow rate of 10 μL.min^-1^ with buffer A (5% acetonitrile and 0.1% formic acid) and separated on a reversed phase column (Acclaim PepMap100 C18, 3 μm, 75 mm i.d. × 500 mm) with a linear gradient of 5–50% buffer B (75% acetonitrile and 0.1% formic acid) at a flow rate of 250 nL.min^-1^ and temperature of 45°C. Gradient length was 100 min and 240 min for gel bands and eFASP samples, respectively. The column was washed with 99% of buffer B for 10 min and reconditioned with buffer A. The total time for an LC MS/MS run was about 120 min long for gel band analysis and 270 min for eFASP digestion.

### Mass Spectrometry

The MS data was acquired on a Q-Exactive^TM^ plus instrument (Thermo Scientific^TM^) using a data-dependent top 20 method, dynamically choosing the most abundant precursor ions from the survey scan (350–1600 m/z) for Higher energy Collisional Dissociation (HCD) fragmentation. Dynamic exclusion duration was 60 s. Isolation of precursors was performed with a 1.6 m/z window and MS/MS scans were acquired with a starting mass of 80 m/z. Survey scans were acquired at a resolution of 70,000 at m/z 400 (AGC set to 10^6^ ions with a maximum fill time of 100 ms). Resolution for HCD spectra was set to 70,000 at m/z 200 (AGC set to 10^5^ ions with a maximum fill time of 200 ms). Normalized collision energy was 28 eV. The underfill ratio, which specifies the minimum percentage of the target value likely to be reached at maximum fill time, was defined as 0.4%. The instruments was run with peptide recognition mode (i.e., from 2 to 8 charge), exclusion of singly charged and of unassigned precursor ions enabled.

### Protein Identification With PEAKS 7.0

The acquired raw files were analyzed with PEAKS Studio 7.0 (Bioinformatics Solutions Inc.) (Zhang et al., 2012) using a custom made database including PGSC_DM_v3.4_pep_non-redundant (April 2017, 52.570 entries^1^), and the addition of unannotated and miss annotated known proteins according to (Van Harsselaar et al., 2017). The peptide mass tolerance was set to 10 ppm and 0.01 Da for MS/MS. Variable modifications included were as follows: Oxidation of M, Y, H, deamidation of N, Q, carbamidomethylation of C, phosphorylation of Y, S, T, pyro-G. For high-confidence, peptide threshold was FDR 1%, protein -10lgP > 20 and protein were identified with two peptides.

### Label-Free Quantification With MaxQuant and Perseus

The acquired raw files were analyzed with MaxQuant 1.5.3.30 using the Andromeda search engine (Cox and Mann, 2008; Tyanova et al., 2016a). Proteins were identified by searching MS and MS/MS data of peptides against the UniProt-Solanum tuberosum database (April 2017, 49.664 entries) and PGSC_DM_v3.4_pep_non-redundant (April 2017, 52.570 entries). The precursor mass and fragment mass were identified with an initial mass tolerance of 10 ppm and 20 ppm, respectively. The search included variable modifications of methionine and proline oxidation, asparagine and glutamine deamidation, tyrosine, serine and threonine phosphorylation, lysine acetylation, glutamine to pyroglutamate conversion and fixed modifications of carbamidomethyl cysteine. Minimal peptide length was set to six amino acids and a maximum of three mis-cleavages was allowed. The false discovery rate (FDR) was set to 0.01 for peptide and protein identifications. MS runs were analyzed with the “match between runs” option 2 min and a 30-min retention time window. In the case of identified peptides that are all shared between two proteins, these were combined and reported as one protein group. Proteins matching to the reverse database were filtered out. LFQ intensities for respective protein groups were uploaded in Perseus and analyzed (Tyanova et al., 2016b). We filter out the reverse (decoy) database hits (proteins match again a reversed or scrambled database), the contaminants (proteins match again contaminants database) and the proteins with a localization probability < 0.75. Raw LFQ intensities were logarithmized by Log2. At least three LFQ values per protein group needed to be present for the analysis. To replace non-quantified values with low intensities, data imputation was performed based on normal distribution of LFQ intensities. Proteins were identified with two peptides minimum. Significant interactors were determined using a two-sample analysis *t*-test and multiple sample test with Benjamini-Hochberg FDR at 0.05. Normalization with *Z*-score was determined and hierarchical clustering was constructed with Euclidean distance.

## Results

### Granule-Bound Proteins Related to Starch Metabolism

**FIGURE 1:**
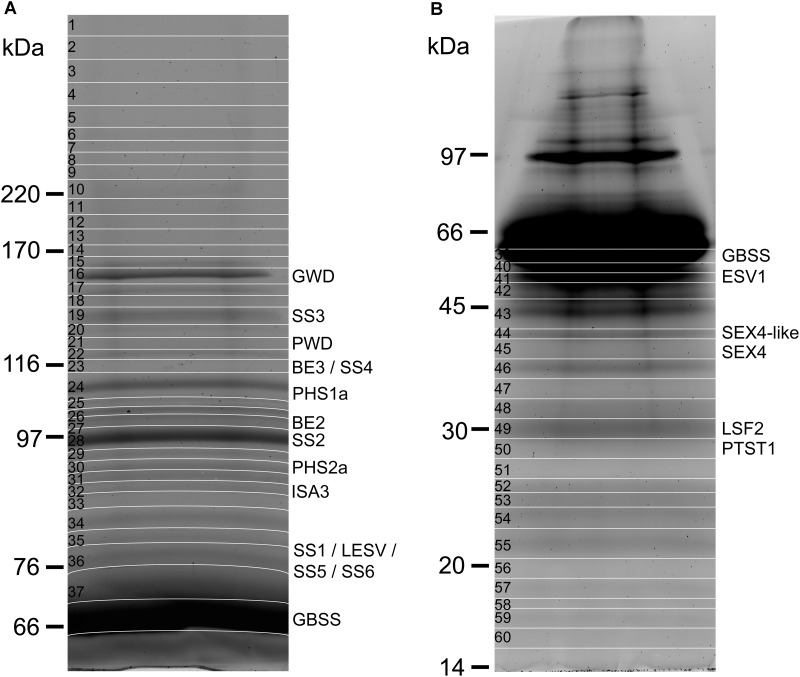
Polyacrylamide-gel electrophoresis of starch-bound proteins from potato starch. **(A)** 300 mg of starch were used for protein isolation. After concentration on an ultrafiltration column, protein samples were loaded on a 7% polyacrylamide gel and submitted to electrophoretic migration at 25 mA during 5 h. The gel was stained with a solution of Sypro ruby (0.5 mg/L) prior to fluorescence imaging. The gel was cut for further analysis according to the pattern displayed on the image (white lines). **(B)** As in **(A)** with the use of a 10% polyacrylamide gel.

Starch-bound proteins were analyzed by SDS-PAGE and fluorescence imaging after isolation from 300 mg of potato starch and concentration by ultrafiltration columns (**Figure 1**). Appropriate separation of bands with an apparent MW above 60 kDa and below 45 kDa was achieved with the use of 7 and 10% polyacrylamide gels, respectively (**Figures 1A,B**). Apparent MW of each band was determined from their relative migration distance (Rf) using the linear range of a standard curve constructed with the Rfs of the protein ladder (**Table 1** and Supplementary Figure S1). Sixty gel bands were cut for further analysis according to the patterns displayed in **Figures 1A,B**. Each band was submitted to in-gel trypsin hydrolysis prior to nanoLC-nanoESI-MS/MS analysis. Protein identification was then carried out with the use of a homemade peptide database including non-redundant CDS annotated by the potato genome sequencing consortium (PGSC^2^) (Hirsch et al., 2014) (Supplementary Data Sheets 1, 2). Several starch metabolism isoforms including BE2 were identified in previous studies but have not been annotated in this database (Van Harsselaar et al., 2017). On the other hand, known starch metabolism genes were miss annotated or annotated under a truncated form (e.g., *SS3*, *PHS1a, ISA3*). Therefore, the iTAG ID, Sotub09g011090.1.1 (BE2), Sotub02g012780.1.1 (AMY3-like), Sotub07g025820.1.1 (BE1.2) and UniProt references, P30924 (BE1), Q43846 (SS3), P32811 (PHS2a), Q84YG5 (ISA3), and P04045 (PHS1a) were manually added to the database. The protein confidence threshold (-10LgP) was empirically set to 100 and proteins were identified with at least two peptides for a confident identification.

**Table 1:** Proteins related to starch metabolism.

Isoform	PGSC/UniProt/iTAG IDs	Predicted MW (kDa)	Observed MW (kDa)	Band number	Number of matched unique peptides	Coverage (%)
GWD	PGSC0003DMP400013565	163.2	160.7	17	78	39
SS3	Q43846	139	142.7	19	57	37
PWD	PGSC0003DMP400029040	132.1	129.3	21	27	25
SS4	PGSC0003DMP400014590	113.8	122.9	23	4	4
BE3	PGSC0003DMP400017627	104.1	122.9	23	9	9
PHS1a	P04045	109.4	118.2	24	23	28
BE2	Sotub09g011090.1.1	100	105.7	27	54	41
SS2	PGSC0003DMP400002384	85.2	103.0	28	203	87
PHS2a	P32811	95.1	96	30	15	21
ISA3	Q84YG5	87.4	88.9	32	31	45
SS1	PGSC0003DMP400032329	70.6	74.1	36	46	46
LESV	PGSC0003DMP400028510	63.4	74.1	36	72	77
SS6	PGSC0003DMP400023948	69.5	74.1	36	9	29
SS5	PGSC0003DMP400053324	76.5	74.1	36	10	27
GBSS1	PGSC0003DMP400021398	66.5	62.8	39	870	94
ESV1	PGSC0003DMP400017605	48.9	51.8	41	6	17
SEX4-like	PGSC0003DMP400047519	41.5	42.8	44	12	38
SEX4	PGSC0003DMP400026742	41.5	38.3	45	11	33
LSF2	PGSC0003DMP400050640	24.9	30.7	49	13	50
PTST1	PGSC0003DMP400053308	28.5	28.6	50	21	58


*Observed MW was estimated from the relative migration distance (Rf) of each band with the use of a calibration curve constructed with the Rfs of the protein ladder.*

**FIGURE 2:**
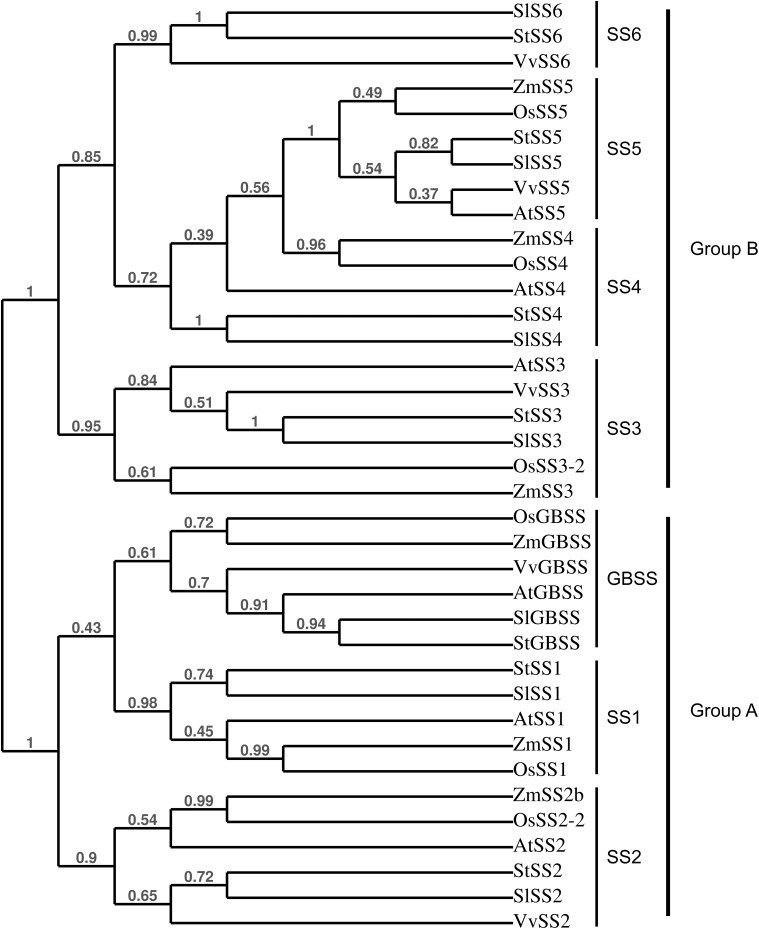
Phylogenetical tree showing the relationships between plant SSs. The tree was constructed with the use of PhyML (Maximum-likelihood based) and confidence limits were assigned by bootstrapping the alignment with 100 trials. At, *Arabidopsis thaliana*; St, *Solanum tuberosum*; Zm, *Zea maize*; Os, *Oryza sativa*; Vv, *Vitis vinifera*; Sl, *Solanum lycopersicum*. SlSS6: NP_001234387.1, StSS6: XP_006353746.1, VvSS6: NP_001268021.1, ZmSS5: NP_001123603.1, OsSS5: XP_015626202.1, StSS5: PGSC0003DMP400053324, SlSS5: XP_019067616.1, VvSS5: NP_001290013.1, AtSS5: NP_569018.1, ZmSS4: NP_001123590.1, OsSS4: XP_015639005.1, AtSS4: NP_193558.3, StSS4: PGSC0003DMP400014590, SlSS4: NP_001234617.2, AtSS3: NP_001184965.1, VvSS3: XP_002269011.2, StSS3: Q43846, SlSS3: NP_001234623.1, OsSS3-2: XP_015650668.1, ZmSS3: NP_001104881.2, OsGBSS: XP_015644490.1, ZmGBSS: NP_001105001.3, VvGBSS: XP_010660257.1, AtGBSS: NP_174566.1, SlGBSS: NP_001311458.1, StGBSS: PGSC0003DMP400021398, StSS1: PGSC0003DMP400032328, SlSS1: XP_010318024.1, AtSS1: NP_197818.1, ZmSS1: NP_001104892.1, OsSS1: XP_015644241.1, ZmSS2b: NP_001106014.1, OsSS2-2: XP_015627452.1, AtSS2: NP_186767.1, StSS2: PGSC0003DMP400002383, SlSS2: XP_004232219.1, VvSS2: XP_010661072.1.

**FIGURE 3:**
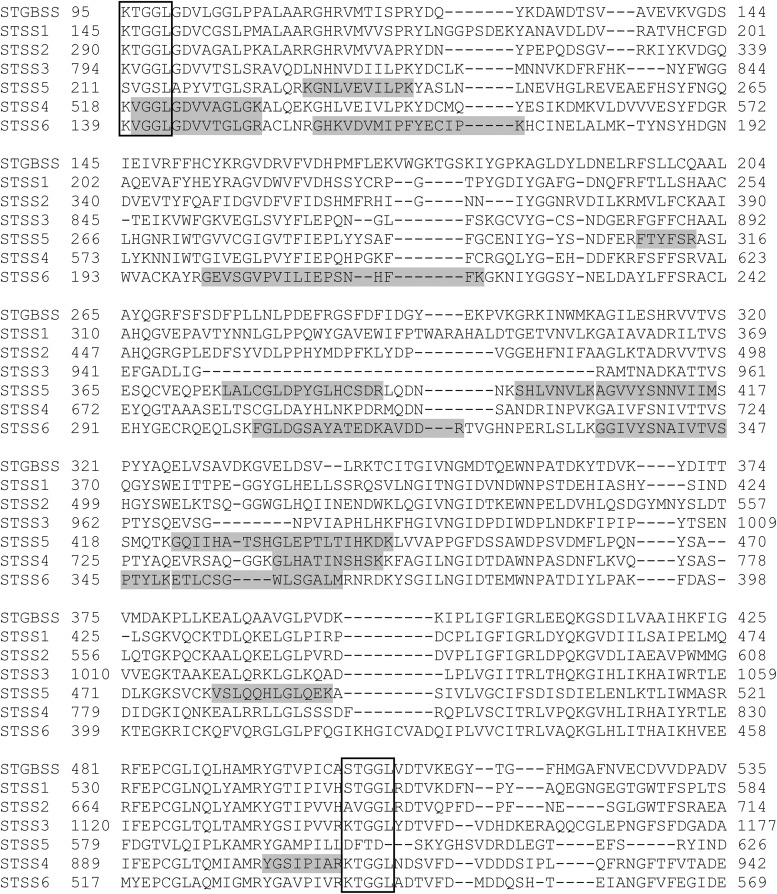
Sequence alignment of the catalytic domains of starch synthase proteins from *Solanum tuberosum*. The protein alignment was constructed with the use of clustal omega (https://www.ebi.ac.uk/Tools/msa/clustalo/). The outlined boxes indicate the highly conserved K-X-G-G-L and X-X-G-G-L motifs. The unique peptides identified by mass spectrometry are highlighted in gray within SS4, SS5, and SS6 sequences. St, *Solanum tuberosum*, StGBSS: PGSC0003DMP400021398, StSS1: PGSC0003DMP400032328, StSS3: Q43846, StSS4: PGSC0003DMP400014590, StSS5: XP_006357749.1, StSS6: XP_006353746.1.

A total of 36 proteins were identified in this study (**Tables 1**, **2** and Supplementary Data Sheets 1, 2), 20 of which were related to starch metabolism (**Table 1**). GBSS is the major starch-binding protein (Stensballe et al., 2008). According to previous descriptions, a band of high intensity was observed at 62.8 kDa, similar to the predicted MW of potato GBSS (**Figures 1A,B** and **Table 1**). In addition, while only SS2 and GWD were previously detected in association with potato starch, SS1, SS2, SS3, BE1, BE2, GWD, and PHS1 have been described in maize, wheat, rice, pea, and barley (Grimaud et al., 2008; Stensballe et al., 2008). These isoforms were also found in our study (**Table 1**). Noteworthy, we observed two additional isoforms of SS, SS5 and SS6, with 10 and 9 unique peptides, respectively, as well as a protein highly homologous to SEX4 annotated as SEX4-like (**Table 1**) (Van Harsselaar et al., 2017). Both *SS5* and *SS6* genes were annotated in potato but their functions have not yet been investigated (Deschamps et al., 2008; Yu et al., 2012; Liu et al., 2015; Van Harsselaar et al., 2017). BLAST search^3^ with the use of the potato SS6 sequence, XP_006353746.1, reveals two other genes similarly annotated in *Solanum lycopersicum* (NP_001234387.1) and *Vitis vinifera* (NP_001268021.1). Furthermore, this analysis showed 90 sequences of hypothetical proteins with a score ranging from 572 to 1301 and an *E*-value of 0 in the genomes of numerous plant species, indicating that the gene is conserved. Phylogenetic analysis including all classes of SSs confirmed that SS6 proteins define a new class of starch synthases (**Figure 2**). SS6 class belongs to group B-starch synthases similar to SS3-5, and most closely resembles to SS4 while the protein lacks the long N-terminal extension usually observed in SS4 proteins (Leterrier et al., 2008). Moreover, the SS6 sequence contains both K-X-G-G-L and X-X-G-G-L highly conserved motifs, whereas the latter are missing in SS5 (**Figure 3**) (Cao et al., 1999).

**Table 2:** Other proteins identified in this study.

PGSC ID	Protein annotation	Predicted MW (kDa)	Number of matched unique peptides	Coverage (%)
PGSC0003DMP400016823	Kunitz-type proteinase inhibitor	20.1	5	35
PGSC0003DMP400017933	Serine protease inhibitor 7	24	7	35
PGSC0003DMP400017950	Cysteine protease inhibitor 1	24.8	5	37
PGSC0003DMP400017953	Kunitz-type tuber invertase inhibitor	24.5	16	67
PGSC0003DMP400017939	Cysteine protease inhibitor 1	21.8	10	26
PGSC0003DMP400016824	Aspartic protease inhibitor 5	23.9	2	22
PGSC0003DMP400008029	Proteinase inhibitor type-2 P303.51	16.6	11	63
PGSC0003DMP400017942	Cysteine protease inhibitor 1	25.1	2	15
PGSC0003DMP400017952	Cysteine protease inhibitor 9	13.8	6	66
PGSC0003DMP400016822	Aspartic protease inhibitor 8	24.1	3	52
PGSC0003DMP400021964	Thioredoxin	19.5	4	38
PGSC0003DMP400028845	Stem 28 kDa glycoprotein	29.5	5	26
PGSC0003DMP400045625	Glutathione peroxidase	26.1	7	33
PGSC0003DMP400009292	Peptidyl-prolyl *cis*–*trans* isomerase	26.5	8	29
PGSC0003DMP400011774	Ci21A protein	12.4	4	30
PGSC0003DMP400051081	Beta-tubulin	50.5	2	16


Two isoforms of PHS2, PHS2a and PHS2b, were identified in the potato genome (Van Harsselaar et al., 2017). This enzyme is also called PHSH (High glycogen activity) and was reported as a cytosolic form of starch phosphorylase participating to the catabolism of starch degradation products in the cytosol (Fettke et al., 2004, 2005). On the other hand, the protein was also identified in proteomic studies of the plastid stroma, suggesting multiple sub-cellular localization for this enzyme (Zybailov et al., 2008; Helm et al., 2014). Interestingly, PHS2a was found attached to the starch granule in this study (**Table 1**). Moreover, PTST1, a protein responsible for targeting GBSS to the starch granule, as well as ESV1 and LESV, two regulatory proteins involved in starch degradation through unknown mechanisms, were recently identified in Arabidopsis (Seung et al., 2015; Feike et al., 2016). The potato genome contains homologs of PTST1, LESV, and ESV1 that were observed in our analysis at 28.6, 74.1, and 51.8 kDa, respectively, which is in good accordance with their predicted MW (**Figure 3** and **Table 1**). Among starch degradation enzymes, PWD, SEX4, ISA3, and LSF2 were detected with a number of unique peptides ranging from 11 to 31 (**Table 1**), thus corroborating that these enzymes interact with starch granules.

### Other Starch-Bound Proteins

In addition to proteins related to starch metabolism, we identified 10 sequences of predicted protease inhibitors (**Table 2**). Protein sequence alignment and phylogenetic analysis established a classification comprising four groups within which the level of protein identity ranges from 77.55 to 99.09% (Supplementary Figure S2A). Nine of these proteins belonged to the potato Kunitz-type proteinase inhibitor (PKPI) family and grouped specifically with one of the three PKPI classes, A, B or C (Ishikawa et al., 1994) (Supplementary Figure S2B). The two other proteinase inhibitors contained 2 or 3 proteinase inhibitor II (PINII) domains and belonged to the potato type II protease inhibitor family (Supplementary Figure S2). Intriguingly, this analysis also revealed the presence of a thioredoxin (TRX) protein (PGSC0003DMP400021964) homologous to Arabidopsis thioredoxins M as well as a glutathione peroxidase (GPX, PGSC0003DMP400045625) similar to Arabidopsis GPX1 (**Table 2** and Supplementary Data Sheet S2). Both enzymes are involved in redox regulation in plants with TRXM participating to the regulation of metabolic processes in function of light and GPX acting during plant-cell stress response (Buchanan, 1984; Noctor et al., 2012). Additionally, an acid phosphatase (PGSC0003DMP400028845, stem 28 kDa glycoprotein) was identified with five unique peptides and a Peptidyl-prolyl *cis*–*trans* isomerase (PGSC0003DMP400009292) belonging to the cyclophilin family with eight unique peptides (**Table 2**). Finally, four unique peptides of a Ci21A protein as well as two peptide from b-tubulin were found in this study (**Table 2**).

### Surface Treatment of the Starch Granules

**FIGURE 4:**
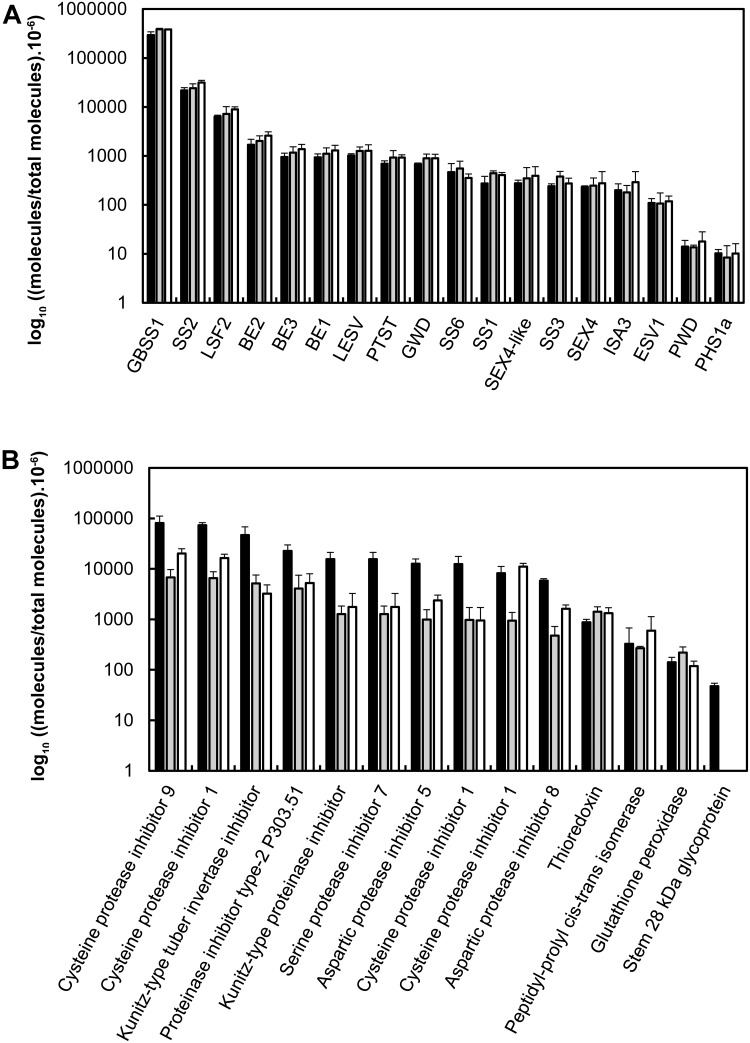
Label free quantification of proteins following SDS- or thermolysin- treatment of starch granules. **(A)** Proteins were isolated from starch prior to nanoLC-MS/MS shotgun analysis of the trypsin digests. The amount of each starch metabolic protein was estimated with the use of the MaxQuant software and the number of observed molecules/total number of predicted molecules was plotted on a log-10 scale. Black bars: non-treated starch, gray bars: SDS treatment, open bars: thermolysin treatment. **(B)** The other proteins identified in this study were quantified and plotted as described in **(A)**.

To investigate the nature of protein interactions with the starch granule, the samples were submitted to SDS-washes or thermolysin treatment prior to protein isolation and MS/MS shotgun analysis after eFASP tryptic digestion (**Figure 4**). Proteins were quantified by the MaxLFQ procedure with the use of the MaxQuant software (Cox et al., 2014) (**Figure 4** and Supplemtary Data Sheet S3). As expected, label-free quantification of starch-bound proteins confirmed that GBSS is the predominant isoform, representing 87–69% of total proteins in non-treated and SDS-treated samples, respectively (**Figure 4A**). This disparity was due to a significant decrease ranging from 75 to 92% in the content of the protease inhibitors following surface treatment whereas all starch metabolism proteins including GBSS remained constant (**Figures 4A,B**). These data allow building two groups according to protein behaviors following surface treatment of starch granules. A drastic decrease was observed in the first group including protease inhibitors and the acid phosphatase indicating weak interaction of these proteins with starch polymers (**Figure 4B**). On the other hand, the second group comprised the enzymes of the starch metabolic pathway that remained attached to starch (**Figure 4A**). Interestingly, the thioredoxin, cyclophilin and the glutathione peroxidase displayed a profile similar to that of starch biosynthetic enzymes, suggesting that their interaction with starch is of the same nature.

## Discussion

The main objective of this study was to investigate exhaustively the proteome associated with potato starch granules. This work relied on the publication of the potato genome (Xu et al., 2011) as well as on a previous genomic analysis of potato genes related to starch metabolism (Van Harsselaar et al., 2017). In addition to the 77 loci identified in the latter study, bottom-up proteomics including *in silico* analysis of all predicted genes allowed us to seek for new, previously uncharacterized starch-bound proteins. Our data confirm previous findings of GBSS, SS2, and GWD as the major proteins bound to potato starch (Stensballe et al., 2008). In addition, we report that SS1, SS3, BE1, BE2, and PHS1a, already observed in other plant species, are also found in association with starch in potato (Grimaud et al., 2008). This result is particularly striking since SSs, BEs as well as PHS1 are known to form protein complexes that are likely modulating individual protein activities (Tetlow et al., 2004, 2008a). Our study lends support both on the conservation of these protein-protein interactions in potato and the interaction of these complexes with the granule. Technical limitations did not allow investigating protein complexes from starch-isolated proteins. Studies on protein–protein complexes usually examine the soluble fraction (i.e., stromal proteins) since physicochemical treatments applied during the process of starch-bound protein extraction alters protein-protein interactions. Apart from GBSS, it is not yet clear, *in planta*, whether starch synthesizing enzymes are active under their soluble forms, when physically bound to the granule, or both. Developing milder extraction methods for the isolation of the starch-bound proteome would contribute answering this question by providing information on the maintenance of protein–protein complexes within the starch matrix.

Recent investigation showed that GBSS is actively targeted to starch granules by PTST1 through reciprocal coiled-coil interaction and glucan binding via a carbohydrate binding module (CBM) present in the N-terminal region of PTST1 (Seung et al., 2015). PTST1 was identified in our analysis thus confirming physical interaction with starch in potato. The question whether PTST1 interacts with GBSS in potato tubers as well as with other starch metabolic proteins arises and requires further investigation. Several other starch-binding proteins contain coiled-coil domains that could drive physical association with PTST1. Noteworthy, this is the case of SS4 that was recently shown to interact with PTST2, a protein homologous to PTST1 and an ortholog of the rice FLO6, in Arabidopsis leaves (Seung et al., 2017). The proposed function of PTST2 is redundant with that of PTST3 for binding and delivering maltooligosaccharides to SS4 during starch initiation (Seung et al., 2017). These findings point out that protein-protein and protein–glucan interactions are actively controlled both at the steps of starch initiation and granule growth. Interestingly, whereas traces of SS4 were detected in the present work, neither PTST2/FLO6 nor PTST3 orthologs were observed. However, plastid physiology, and likely starch metabolism, are substantially different between Arabidopsis leaf chloroplasts and potato tuber amyloplasts. Moreover, while the function of SS4 in the initiation and the control of starch granule number have been studied extensively in Arabidopsis, its function in storage starch synthesis of the potato tuber remains to be investigated.

Proteins of the starch degradation pathway were also identified in this work. These comprise PWD, GWD, SEX4, ISA3, LSF2 as well as the recently discovered degradation regulators ESV1 and LESV. Interaction of PWD and GWD with the starch granule is well documented (Ritte et al., 2000; Kötting et al., 2005; Stensballe et al., 2008). Similarly, the present work confirms that both SEX4 and LSF2, already observed in association with Arabidopsis starch, bind to the starch granules in potato tubers (Santelia et al., 2011). On the other hand, to our knowledge, this is the first report on the physical association of ISA3 with starch. Like ISA1, ISA3 hydrolyzes α-1,6 linkages of amylopectin. However, whereas ISA1 participate to starch synthesis in complex with ISA2, ISA3 is involved in transitory starch breakdown in leaves of Arabidopsis and rice (Wattebled et al., 2005; Yun et al., 2011). The function of ISA3 in amyloplasts is not yet fully elucidated as illustrated by the phenotype of an *isa3* mutant of rice (Yun et al., 2011). In the latter, plastid morphogenesis is altered as well as the morphology of starch granules. The authors proposed that ISA3 impacts plastid division in rice leading to pleomorphic amyloplasts and starch granules when the gene is knocked out (Yun et al., 2011). Simultaneous antisense inhibition of ISA1, ISA2, and ISA3 in potato do not alter leaf starch metabolism (Ferreira et al., 2017). On the other hand, starch accumulation in tubers of the latter lines is significantly reduced (Ferreira et al., 2017). Furthermore, granule morphology is altered with an increase in the proportion of the smallest starch granules, which was already observed in antisense lines where the expression of only *ISA1* and *ISA2* is reduced (Bustos et al., 2004; Ferreira et al., 2017). Thus, while the function of the ISA1/ISA2 complex in starch synthesis in plants including potato is supported by several lines of evidence, elucidating that of ISA3 would require further studies such as the characterization of knock out mutants (Hussain et al., 2003). One possible explanation for the presence of ISA3 within the starch granule is that the protein interacts with a CBM-containing protein similar to ISA1 interacting with FLO6 in the rice endosperm (Peng et al., 2014). The authors proposed that while not directly binding to starch, rice ISA1 binds to FLO6 which, in turn, bridges between starch and the enzyme (Peng et al., 2014).

The finding of an isoform of PHS2, usually referred to as the cytosolic starch phosphorylase, in the present analysis was intriguing. This starch phosphorylase is strongly interacting with a complex cytosolic heteroglycan in several plant species comprising potato (Yang and Steup, 1990; Fettke et al., 2008). This heteroglycan is a glucosyl acceptor for PHS2 activity in starch catabolism consistent with a cytosolic localization of the enzyme, which was confirmed by immunofluorescence studies of pea leaves as well as potato leaves overexpressing PHS2 from *Vicia faba* (Conrads et al., 1986; Fettke et al., 2004, 2005). On the other hand, the protein was identified in the chloroplast stroma of Arabidopsis by proteomics, suggesting a dual-targeting of the enzyme (Zybailov et al., 2008; Helm et al., 2014). Interestingly, contrary to Arabidopsis, the potato genome contains two isoforms of PHS2, namely, PHS2a and PHS2b (Van Harsselaar et al., 2017). Our observation of starch-bound PHS2a corroborates that this isoform is not restricted to the cytosol. However, it is also possible that the enzyme, known to strongly interact with polysaccharides was copurified with starch during the experimental procedure. Unfortunately, our MaxLFQ analysis following surface treatment of starch did not allow quantifying this particular isoform and consequently to investigate the type of interaction between the enzyme and the starch granule.

Identification of the two isoforms of starch synthase, SS5 and SS6, in this study was particularly fascinating. Indeed, while both isoforms were already observed in the genomes of numerous plant species, evidence for their participation to starch metabolism are sporadic (Deschamps et al., 2008; Yu et al., 2012; Liu et al., 2015; Van Harsselaar et al., 2017). These only relied on the gene expression profiles during grain filling in maize and potato tuberization for SS5 and SS6, respectively (Yu et al., 2012; Liu et al., 2015). Our study is the first report on the presence of these isoforms in association with the starch granule, arguing for a function in starch metabolism. Phylogenetic analysis showed that both proteins have a monophyletic origin and belong to Group-B starch synthase similar to SS3 and SS4 (Leterrier et al., 2008). Although a detailed phylogenomic analysis of *SS6* remains to be performed, the gene appears to be conserved among dicots with the noticeable exception of Arabidopsis. The Arabidopsis genome contains a truncated version of *SS5* and no *SS6* gene was observed in this species. The potato SS5 sequence lacks the two X-X-G-G-L motifs suggesting that this protein is not catalytically active. However, one cannot exclude that this isoform diverged functionally during plant evolution and carries a specific enzymatic activity that remains to be characterized. On the other hand, it is also possible that, while devoid of any enzymatic activity, the protein is responsible for the maintenance of protein–protein, protein–glucan, or glucan–glucan interactions, as suggested for ESV1 and LESV (Feike et al., 2016). Contrarily to SS5, SS6 contains the catalytic amino acid residues of starch synthases suggesting a function in the elongation of α-1,4 starch polysaccharides (Cao et al., 1999). This isoform is most closely related to SS4, which is involved in starch initiation and the control of granule number and shape in Arabidopsis (Roldan et al., 2007). Interestingly, SS6 differs from SS4 by the absence of the long N-terminal extension but both proteins share the catalytic glucosyltransferase domain. The former is known to modulate the subcellular localization of SS4 as well as conditioning granule shape while the latter is responsible for starch initiation, thus controlling the number of starch granules in Arabidopsis (Raynaud et al., 2016; Lu et al., 2017). Potato amyloplasts contain a single, relatively huge, spherical starch granule, similar to *ss4-* leaf chloroplasts when compared to wild type plants (usually accumulating 5–7 granules per chloroplast) (Roldan et al., 2007). It is thus tempting to speculate that the function of SS6 supplants that of SS4 in starch initiation in potato tubers leading to the initiation of a single, rounded-shape starch granule, analogous to those of Arabidopsis *ss4-* mutants. Nevertheless, deciphering the functions of SS5 and SS6 will require further investigations including the phenotypic analysis of mutant plants.

In addition to starch metabolic enzymes, the present study revealed the presence of several protease inhibitors belonging to the PKPI or PINII family. The PKPI family comprises three groups, namely A, B, and C. PKPI-A group contains inhibitors of aspartic proteases and chymotrypsin while PKPI-B group includes dual inhibitors of serine proteases, trypsin and chymotrypsin (Heibges et al., 2003). On the other hand, PKPI-C group comprises inhibitors of cysteine proteases and inhibitors of the invertase (Heibges et al., 2003; Poltronieri et al., 2012). However, a high functional polymorphism between PKPIs regardless of the group they belong to was previously suggested (Heibges et al., 2003). On the other hand, PINII proteins are serine protease inhibitors containing eight cysteine residues forming disulfide bounds and are involved in plant defense against pathogens (Turra and Lorito, 2011). Noteworthy, the contents of these proteins were drastically reduced after treating starch granules with SDS or thermolysin indicating weak interaction with starch polysaccharides. These protease inhibitors may either specifically interact with starch or being co-purified during granule isolation. In the former case, one could hypothesize that they protect starch-bound hydrolyzing enzymes from proteolysis for efficient starch degradation concomitant with tuber sprouting. However, the nature of the interaction between starch and the protease inhibitors remains to be characterized, which could help elucidating their potential function in starch metabolism.

Contrary to the protease inhibitors, all starch metabolism proteins that could be quantified in this study remained attached to starch treated with SDS or thermolysin. This result indicate that these proteins are entrapped within the starch matrix. This was also the case of GPX, a glutathione peroxidase and TRX, a thioredoxin protein homologous to Arabidopsis TRX M. The latter protein is involved in the ferredoxin/thioredoxin pathway that regulates metabolic processes in function of light (Buchanan, 1984). Thioredoxin M4 was shown to participate to the redox regulation of starch metabolism in Arabidopsis by activating SS1 and BAM1 (Valerio et al., 2010; Skryhan et al., 2015). On the other hand, glutathione peroxidases are thought to contribute to cellular redox homeostasis during plant-cell stress responses (Noctor et al., 2012). Overall, both proteins are involved in redox regulation of enzyme activities. Their presence in the starch granule suggests that they play a role in regulating starch metabolism and open new perspectives to studying these aspects in storage organs of one of the most cultivated crops.

## Author Contributions

AC and MF cultivated and harvested plants. JV and SD prepared the samples. SH and FB performed the experiments and analyzed the data. NS, CT, and CR conceived and designed the study. NS wrote the manuscript.

## Conflict of Interest Statement

The authors declare that the research was conducted in the absence of any commercial or financial relationships that could be construed as a potential conflict of interest.

## Footnotes

1 http://solanaceae.plantbiology.msu.edu/pgsc_download.shtml

2 http://solanaceae.plantbiology.msu.edu/pgsc_download.shtml

3 https://blast.ncbi.nlm.nih.gov/Blast.cgi
