# Supplementary material for: Proteome Analysis of Potato Starch Reveals the Presence of New Starch Metabolic Proteins as Well as Multiple Protease Inhibitors
Source: Front Plant Sci. 2018 Jun 15;9:746. doi: 10.3389/fpls.2018.00746 (PMC6013586; doi:10.3389/fpls.2018.00746)
Supplement: Supplementary file 1 [file Presentation_1.pdf]

Supplementary Material

Proteome analysis of potato starch reveals the presence of new starch metabolic proteins as well as multiple protease inhibitors

Stanislas Helle[1,2*], Fabrice Bray[2*], Jérémy Verbeke[2], Stéphanie Devassine[2], Adeline Courseaux[1], Maud Facon[1,2], Caroline Tokarski[2], Christian Rolando[2], Nicolas Szydlowski[1,2†]

[1] Univ. Lille, CNRS, UMR8576 – UGSF – Unité de Glycobiologie Structurale et Fonctionnelle, F-59000 Lille, France

[2] Univ. Lille, CNRS, USR 3290 – MSAP – Miniaturisation pour la Synthèse l'Analyse et la Protéomique, F-59000 Lille, France

***** equal contribution

[†] **Correspondence:**
Nicolas Szydlowski
nicolas.szydlowski@univ-lille1.fr

1 Supplementary data

Supplementary Data Sheet 1. LC-MS/MS analysis of the bands excised from a 7% polyacrylamide gel after electrophoretic migration of starch-isolated proteins. The first and second tabs provide information on column titles and protein accession numbers, respectively. The following tabs are numbered according to band numbers.

Supplementary Data Sheet 2. LC-MS/MS analysis of the bands excised from a 10% polyacrylamide gel after electrophoretic migration of starch-isolated proteins. The first and second tabs provide information on column titles and protein accession numbers, respectively. The following tabs are numbered according to band numbers.

Supplementary Data Sheet 3. Label-free quantification of proteins following SDS or thermolysin treatment with Maxquant. The first tab describes the column titles.

2 Supplementary Figures

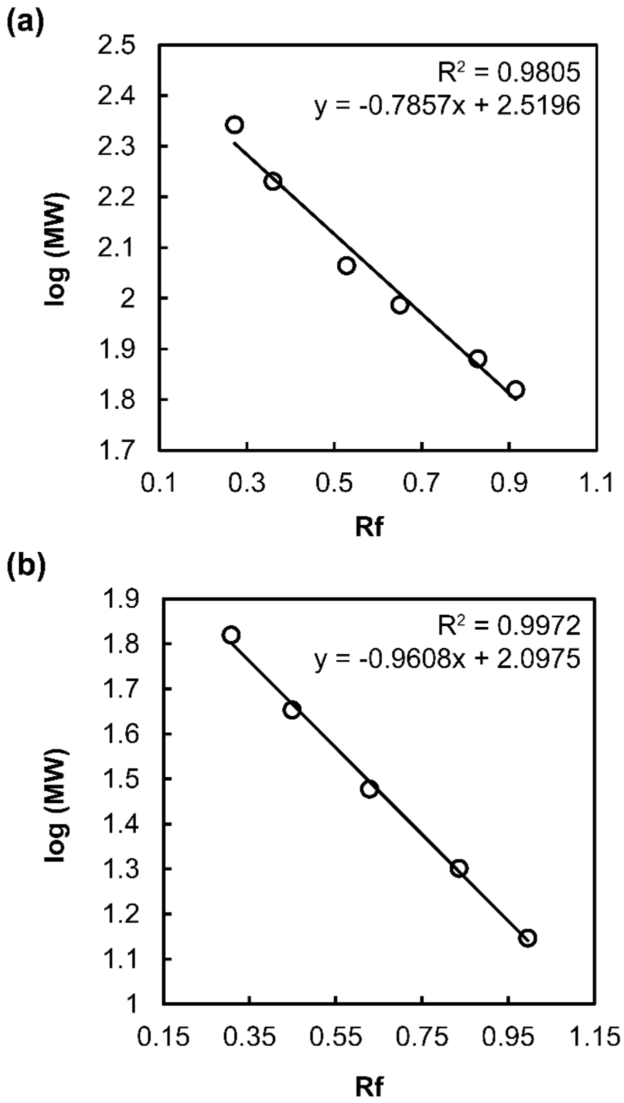

Supplementary Figure 1. Standard curves for protein ladder molecular weights.

(a) After electrophoretic migration on a 7% polyacrylamide gel and fluorescence staining, the Rfs of the protein ladder bands (220 kDa, 170 kDa, 116 kDa, 97 kDa, 76 kDa, 66 kDa) were determined. The migration distance of each band and of the dye front were measured from the top of the resolving gel with the use of Fiji (Schindelin et al., 2012;Schneider et al., 2012) and Rfs were plotted versus log (MW).

(b) As in (a) with the use of a 10% polyacrylamide gel and the Rfs from the protein ladder bands: 66 kDa, 45 kDa, 30 kDa, 20 kDa and 14 kDa.

(a)

	PGSC0003DMP400008029	PGSC0003DMP400008030	PGSC0003DMP400026811	PGSC0003DMP400017939	PGSC0003DMP400017953	PGSC0003DMP400017952	PGSC0003DMP400017950	PGSC0003DMP400017944	PGSC0003DMP400016823	PGSC0003DMP400017933	PGSC0003DMP400016824	PGSC0003DMP400016822
**PGSC0003DMP400008029**	100											
**PGSC0003DMP400008030**	85.71	100										
**PGSC0003DMP400026811**	81.81	91.21	100									
**PGSC0003DMP400017939**	12.98	15.3	14.79	100								
**PGSC0003DMP400017953**	14.93	16.09	15.12	78.06	100							
**PGSC0003DMP400017952**	5.69	13,00	12.19	82.11	88.61	100						
**PGSC0003DMP400017950**	15.58	16.09	15.12	78.06	92.69	93.49	100					
**PGSC0003DMP400017944**	14.93	15.6	14.63	77.55	91.78	92.68	99.09	100				
**PGSC0003DMP400016823**	11.03	11.29	10.75	25.8	33.87	24.39	34.4	34.4	100			
**PGSC0003DMP400017933**	11.03	12.68	12.19	28.06	32.87	31.7	33.03	33.48	95.16	100		
**PGSC0003DMP400016824**	11.68	13.17	13.17	27.04	31.19	29.26	31.65	32.56	73.11	73.85	100	
**PGSC0003DMP400016822**	11.68	13.17	13.17	26.02	30.59	27.64	31.05	31.96	72.04	74.42	91.28	100

(b)

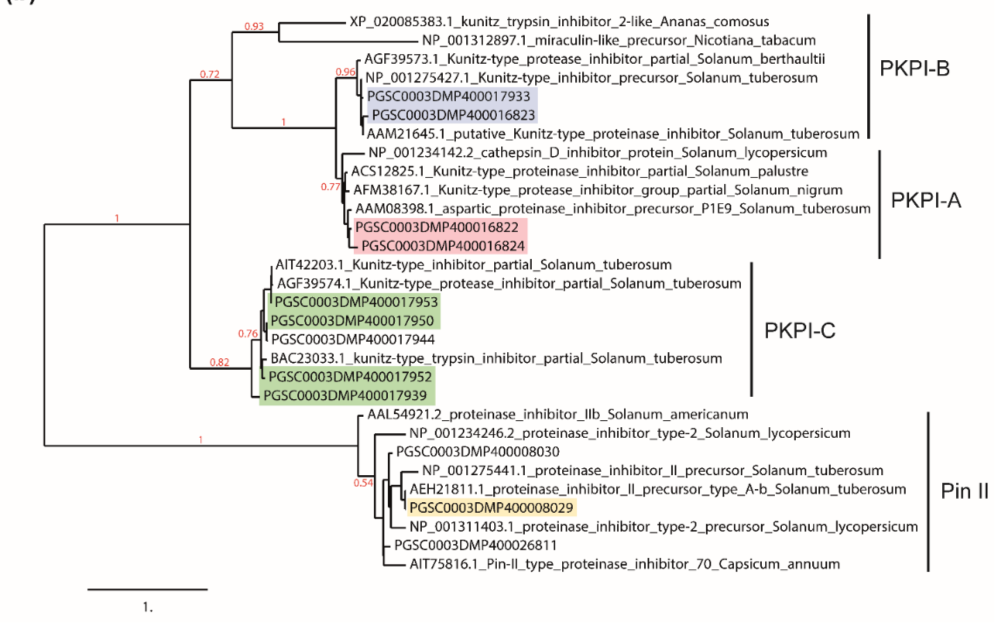

Supplementary Figure 2. Sequence identity and phylogenetic analysis of potato proteinase inhibitors.

(a) Proteinase inhibitor sequences were aligned with the use of clustal omega (https://www.ebi.ac.uk/Tools/msa/clustalo/) and the identity percentages were calculated with SIAS (http://imed.med.ucm.es/Tools/sias.html). For identity calculation, the sequence length was normalized to the smallest sequence.

(b) The tree was constructed with the use of PhyML (Maximum-likelihood based) and confidence limits were assigned by Approximate Likelihood-Ratio Test (aLRT). Sequence identifiers are indicated on the tree.
